# Associations of accelerometer‐based sedentary time, light physical activity and moderate‐to‐vigorous physical activity with resting cardiac structure and function in adolescents according to sex, fat mass, lean mass, BMI, and hypertensive status

**DOI:** 10.1111/sms.14365

**Published:** 2023-04-10

**Authors:** Andrew O. Agbaje

**Affiliations:** ^1^ Institute of Public Health and Clinical Nutrition, School of Medicine, Faculty of Health Sciences University of Eastern Finland Kuopio Finland; ^2^ Children's Health and Exercise Research Centre, Department of Public Health and Sports Sciences, Faculty of Health and Life Sciences University of Exeter Exeter UK

**Keywords:** cardiac remodeling, exercise, left ventricular hypertrophy, lifestyle factors, movement behavior, pediatrics

## Abstract

**Background:**

This study examined the independent relationships of device‐based measured sedentary time (ST) and physical activity (PA) in relation to cardiac structural and functional geometry among adolescents.

**Methods:**

From the Avon Longitudinal Study of Parents and Children, UK birth cohort, 530 (50% female) adolescents aged 17 years had complete ST, PA, cardiac, and covariate measures. Echocardiography cardiac measures were left ventricular mass indexed for height^2.7^ (LVMI^2.7^), relative wall thickness, LV diastolic function (LVDF), and LV filling pressure (LVFP). Overweight/obesity and elevated systolic/BP hypertension were categorized as body mass index >24.99 kg/m^2^ and ≥130 mmHg, respectively. Data were analyzed with linear regression models adjusting for cardiometabolic factors and lifestyle factors.

**Results:**

The prevalence of overweight/obesity in males and females was 17.9% and 24.5%, respectively. The prevalence of elevated systolic BP/hypertension was 11.6% in males and 1.1% among females. The average ST was 484 ± 78 min/day, light PA was 274 ± 62 min/day, and moderate‐to‐vigorous PA (MVPA) was 41 ± 24 min/day, among females. Average ST, LPA, and MVPA were 468 ± 87 min/day, 293 ± 70 min/day, and 56 ± 30 min/day, respectively, among males. Higher ST was associated with higher LVMI^2.7^ (standardized *β =* 0.16; *p* = 0.01) among females, but higher ST was associated with lower LVDF in males (*β =* −0.14; *p* = 0.04). Higher ST and MVPA were associated with higher LVMI^2.7^ in the total cohort, normal weight, and overweight/obese adolescents. Light PA was associated with higher LVDF in the total cohort and normotensives and lower LVFP among adolescents with high lean mass.

**Conclusions:**

Higher ST and MVPA were associated with higher LVMI; however, ST‐associated LVMI increase was threefold higher than MVPA‐associated LVMI increase. Higher LPA was associated with better cardiac function. Reducing ST and increasing LPA may attenuate the risk of altered cardiac structure and function in adolescents.

## 1 INTRODUCTION

Abnormal cardiac structure and functional indices are surrogate measures for signs of early cardiac damage in pediatric research.^1^, ^2^, ^3^, ^4^, ^5^ Cardiac structural measures such as left ventricular mass (LVM), relative wall thickness (RWT), and functional measures such as LV diastolic function (LVDF) and LV filling pressure (LVFP) have been associated with clinical endpoints in adults.^6^, ^7^ Elevated blood pressure (BP) and hypertension have been associated with LV hypertrophy and altered LVDF in pediatric population.^2^, ^3^, ^5^ It has been reported that a 5 mmHg increase in pre‐exercise, post‐exercise, and recovery‐exercise systolic BP was associated with higher LVM and RWT in adolescents, but the relationship was significantly attenuated after adjusting for lean mass.^8^ The benefit of increasing physical activity (PA) on health outcomes such as physical fitness, cardiometabolic health (BP, lipid, insulin, and glucose metabolism), bone health, and cognitive function in adolescence is well documented.^9^ A daily 60 min of moderate‐to‐vigorous physical activity (MVPA) has been associated with health benefits but rarely do adolescents meet this daily threshold.^9^, ^10^

There is limited evidence on the independent role of a device‐measured PA on cardiac structure and function in this age group which could improve understanding of physiological adaptation during growth and maturation.^8^, ^9^, ^11^, ^12^, ^13^, ^14^ Moreover, the sex‐specific relationship between sedentary time (ST) and cardiac structural and functional indices in adolescence remains unclear.^15^ Since adolescents accumulate more time engaging in light PA (LPA) than MVPA, it is important to clarify whether LPA and MVPA independently associate with healthier cardiac structure and function in youth.^11^ Similarly, insufficient evidence has limited the quantification of sedentary behavior that associates with a higher risk of precursors of cardiac damage in youth, but higher self‐reported time spent in sedentary behavior has been associated with obesity.^9^, ^11^, ^15^

Therefore, the independent associations of ST, LPA, and MVPA with cardiac structure and function were examined among adolescents based on sex, obesity, body composition, and hypertensive status using data from the Avon Longitudinal Study of Parents and Children (ALSPAC) birth cohort, England, United Kingdom. It was hypothesized that higher ST would associate with poorer cardiac indices while higher LPA and MVPA would associate with better cardiac structure and function.

## 2 METHODS

### 2.1 Study cohort

**FIGURE 1:**
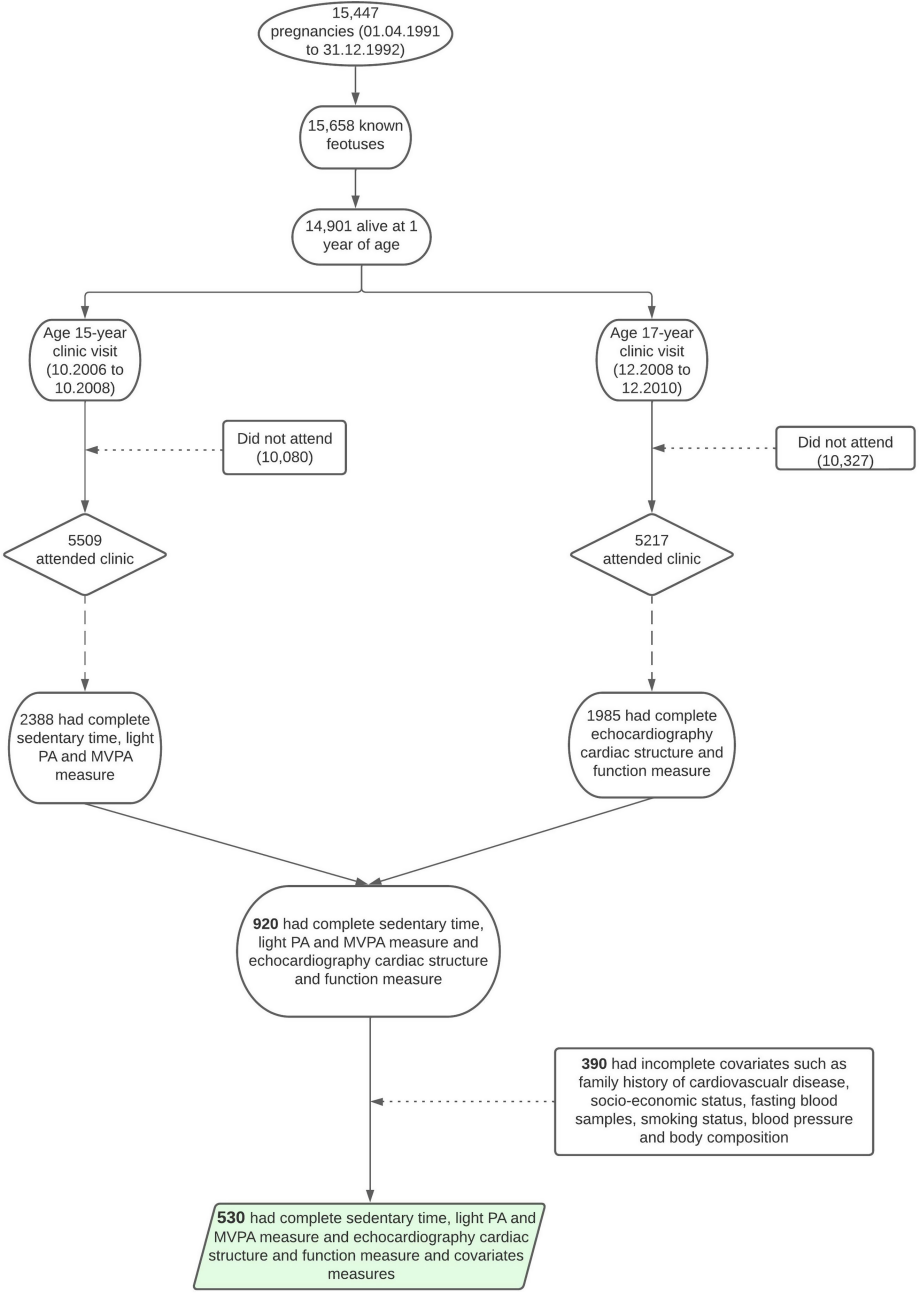
Flowchart of study participants. Light PA, light physical activity; MVPA, moderate‐to‐vigorous physical activity.

Data were from the ALSPAC birth cohort, which investigates factors that influence childhood development and growth. Altogether, 14 541 pregnancies from women residing in Avon, southwestern England, UK, who had a total of 14 676 fetuses, were enrolled between April 1, 1991, and December 31, 1992. When the oldest children were approximately 7 years of age, an attempt was made to bolster the initial sample with eligible cases who had failed to join the study originally resulting in 913 additional pregnancies. The total sample size for analyses using any data collected after 7 years of age is 15 454 pregnancies, resulting in 15 589 fetuses. Of these, 14 901 were alive at 1 year of age.^16^, ^17^, ^18^ Regular clinic visits of the children commenced at 7 years of age and are still ongoing. ALSPAC participants had an accelerometer device worn for 7 days and for the analysis 530 participants who had complete accelerometer valid data, cardiac structure, and function variables as well as all covariates' measurements at 17‐year clinic visit were included (Figure 1). Ethical approval for the study was obtained from the ALSPAC Ethics and Law Committee and the Local Research Ethics Committees. Informed consent for the use of data collected via questionnaires and clinics was obtained from participants. Consent for biological samples has been collected in accordance with the Human Tissue Act (2004).

### 2.2 Anthropometric, cardiometabolic, and lifestyle factors measures

Height and weight of participants at age 17 were assessed and body mass index (BMI) was computed as the ratio of mass to squared height. Overweight/obesity was categorized as BMI ≥24.99 kg/m^2^. Heart rate and systolic and diastolic BP were measured.^19^, ^20^ Participants with ≥130 mmHg systolic BP were categorized as elevated systolic BP/hypertension.^21^ Fasting blood sample was assayed for high‐density lipoprotein cholesterol, triglyceride, insulin, glucose, high‐sensitivity C‐reactive protein, and estimated low‐density lipoprotein cholesterol at age 17 years.^19^, ^20^, ^22^ Total body fat mass and lean mass were assessed using a dual‐energy x‐ray absorptiometry scanner at the clinic visit. Sex‐specific categories of fat mass and lean mass above the 75th percentile were classified as high fat mass and high lean mass.^19^, ^23^ Males' 75th percentile for fat mass was 16.9 kg, and lean mass was 58.9 kg. Females' 75th percentile for fat mass was 26.3 kg, and lean mass was 40.7 kg. Questionnaires to assess smoking behavior were administered during the 17‐year clinic visits. Participants provided a family history of hypertension, diabetes, high cholesterol, and vascular disease at the clinic visit. The participant's mother's socioeconomic status was grouped according to the 1991 British Office of Population and Census Statistics classification.^22^, ^24^, ^25^ Time (years) to age at peak height velocity, an objective measure of pubertal or maturation status without having to rely on physical examination or self‐report, was derived using Superimposition by Translation And Rotation mixed‐effects growth curve analysis in the ALSPAC cohort.^26^ However, all participants had attained puberty by age 17 years based on this objective measure.^22^

### 2.3 Sedentary time and physical activity measures

Sedentary time, LPA, and MVPA at approximately age 16 years were assessed with ActiGraphTM accelerometer.^20^ Participants wore the AM7164 accelerometer (Actigraph) for 7 days during waking hours and remove the device only when showering, bathing, and performing water sports. The device captured body movement in terms of acceleration as a combined function of frequency and intensity. Data were processed using Kinesoft software, version 3.3.75 (Kinesoft), according to established protocol. Data were recorded as counts that resulted from summing postfiltered accelerometer values (raw data at 30 Hz) into 60‐s epoch units. The analysis was restricted to participants with 3 or more days of valid data (≥500 min/day, after excluding intervals of ≥60 min of 0 counts). Activity counts per minute thresholds validated in young people were used to calculate the amount of time spent in ST, LPA, and MVPA; for MVPA, >2296 counts per minute; for LPA, 100–2296 counts per minute; and for ST, 0 – <100 counts per minute.^19^, ^27^, ^28^ The Evenson cutpoint used in stratifying activity threshold has shown the best overall performance across all intensity levels and was suggested as the most appropriate cut point for youth.^28^, ^29^

### 2.4 Cardiac structure and function measures

At 17‐year clinic visit, echocardiography was performed according to American Society of Echocardiography guidelines^30^, ^31^ using an HDI 5000 ultrasound machine (Phillips Healthcare) equipped with a P4‐2 Phased Array ultrasound transducer. Two trained cardiac sonographers measured and analyzed all variables. Pulsed Doppler examination of transmitral flow was recorded from the apical four‐chamber view. For LV measurements, the sample volume was positioned between the mitral annulus and the tips of the mitral leaflets with the position adjusted to maintain the sample volume at an angle as near parallel to transmitral flow as possible with the participant in passive end‐expiration. The peak flow velocity of the early (E) and atrial (A) waves was measured from the three consecutive cardiac cycles displaying the highest measurable velocity profiles. Similar measurements (e′ and a′) were also made at the tricuspid valve. The e′‐wave reflects relaxation in early diastole, and the a′‐wave reflects atrial contraction in late diastole.^6^

Measures of cardiac structure were LVM index (LVMI^2.7^) to allometrically scaled ideal body mass^1^, ^3^, ^32^, ^33^ and RWT computed from septal wall thickness, posterior wall thickness, and LV diastolic diameter. Cardiac structural adaptation occurs due to wall stretch from pressure overload concentric remodeling and volume overload eccentric remodeling.^34^ LV volume, mass, and RWT (mass/volume) are important parameters in distinguishing these adaptations. Alteration in structural adaptation results in concentric hypertrophy with no change in the size of cardiac internal cavity and eccentric hypertrophy with changes in the cardiac internal cavity.^34^ Normal end‐diastolic volume and an increased RWT suggests concentric hypertrophy if LVM is increased, and concentric remodeling if LVM is normal.^34^ High end‐diastolic volume and decreased RWT suggest eccentric hypertrophy if LVM is increased, and eccentric remodeling if LVM is normal.^33^ Measures of cardiac function were LVD function E/A wave ratio (LVDF) and LV filling pressure E/e′ wave ratio (LVFP).^3^, ^6^ LVDF describes different physiological processes that allow the LV to fill with sufficient blood for the body's current needs at a low enough pressure to prevent pulmonary congestion.^6^ At the end of LV contraction, pressure drops abruptly in the LV until the mitral valve opens, and blood flows along a negative pressure gradient toward the apex.^6^ This pressure eventually equilibrates between the left atrium and the LV, resulting in diastasis, ventricular filling, and atrial contraction.^6^ Alteration of this physiologic process may result in LVD dysfunction, a cause of heart failure with preserved ejection fraction that accounts for ≥50% of all heart failure cases.^35^ LVDF is assessed from late diastolic transmitral flow velocity (E/A) ratio and LVFP using the E to early diastolic mitral annular tissue velocity (E/e′) ratio.^6^ The reproducibility of these examinations was assessed by recalling 30 participants and repeating their echocardiographic measurements. The intraclass correlation of repeated measurements ranged from 0.75 to 0.93 (intraobserver) and 0.78 to 0.93 (interobserver).^36^

### 2.5 Statistical analysis

Participants' descriptive characteristics were summarized as means and standard deviation, medians, and interquartile ranges, or frequencies and percentages. Sex differences were explored using independent *t*‐tests, Mann–Whitney *U* tests, Chi‐square tests, or one‐way analysis of covariance for normally distributed, skewed, dichotomous, or multicategory variables, respectively. The normality of variables was assessed, and skewed covariates were logarithmically transformed prior to further analyses. The log‐transformed covariates were triglyceride, insulin, high‐sensitivity C‐reactive protein, total body fat mass, and lean mass. The separate association of ST, LPA, and MVPA with each of LVMI^2.7^, RWT, LVDF, and LVFP was examined using linear regression models. The total cohort analyses were adjusted for the following covariates, sex, family history of hypertension/diabetes/high cholesterol/vascular disease, age, low‐density lipoprotein cholesterol, insulin, triglyceride, high‐sensitivity C‐reactive protein, high‐density lipoprotein cholesterol, heart rate, glucose, systolic BP, fat mass, lean mass, smoking status, and mutually adjusting for ST, LPA, or MVPA depending on the predictor.^20^, ^24^, ^37^, ^38^, ^39^, ^40^ The sex–body mass index, sex–BP, sex–fat mass, and sex–lean mass interaction effect for all cardiac indices were statistically significant (*p* < 0.001). Therefore, sex‐, body mass index‐overweight/obesity, and elevated systolic BP/hypertension stratified results were presented. When the cohort was stratified by a characteristic, for example, sex, such analysis was not adjusted for that specific characteristic. Collinearity diagnoses were performed and accepted results with a variance inflation factor <5. Differences and associations with a two‐sided *p*‐value <0.05 were considered statistically significant with conclusions based on standardized regression coefficient. Analyses involving a sample of 500 ALSPAC participants at 0.8 statistical power, 0.05 alpha, and two‐sided *p*‐value would show a minimum detectable effect size of 0.16 standard deviations if they had relevant exposure for a normally distributed quantitative variable.^41^ All statistical analyses were performed using SPSS statistics software, Version 27.0 (IBM Corp).

## 3 RESULTS

### 3.1 Cohort study characteristics

Altogether 530 (50% female) participants who had complete ST, LPA, MVPA, LVMI^2.7^, RWT, LVDF, and LVFP measurements at the age 17 years clinic visit were studied (Figure 1). Males spent more time in LPA and MVPA and had higher LVMI^2.7^ than females. More time was spent as ST among females than males (Table 1). Males had higher lean mass and lower fat mass than females. The prevalence of overweight/obesity in males and females was 17.9% and 24.5%, respectively. The prevalence of elevated systolic BP/hypertension was 11.6% in males and 1.1% among females. Other participants' characteristics are shown in Table 1.

**TABLE 1:** Descriptive characteristics of adolescents.

Variables	Male (*n* = 266)	Female (*n* = 264)	*p*‐Value
	Mean (SD)	Mean (SD)	
Anthropometry			
Age (years)	17.67 (0.28)	17.67 (0.32)	0.881
Height (m)	1.79 (0.07)	1.66 (0.06)	<0.001
*Weight (kg)	68.95 (13.30)	61.00 (14.20)	<0.0001
Ethnicity—White (*n*, %)	254 (95.4)	254 (96.3)	0.696
Body composition			
*Total fat mass (kg)	10.26 (8.94)	19.80 (10.43)	<0.0001
*Lean mass (kg)	54.55 (8.08)	38.09 (4.39)	<0.0001
*Body mass index (kg/m^2^)	21.37 (3.78)	22.46 (4.89)	<0.001
Overweight/obese (BMI >24.99 kg/m^2^ (*n*, %)	48 (17.9)	65 (24.7)	<0.001
Fasting plasma metabolic indices			
High‐density lipoprotein (mmol/L)	1.21 (0.26)	1.37 (0.32)	<0.001
Low‐density lipoprotein (mmol/L)	2.03 (0.58)	2.21 (0.65)	0.001
*Triglyceride (mmol/L)	0.73 (0.38)	0.73 (0.35)	0.458
Glucose (mmol/L)	5.19 (0.63)	4.93 (0.34)	<0.001
*Insulin (mU/L)	5.94 (3.71)	7.57 (4.13)	<0.001
*High‐sensitivity C‐reactive protein (mg/L)	0.45 (0.88)	0.60 (1.53)	<0.001
Vascular measures			
Heart rate (beat/min)	63 (10)	66 (9)	<0.001
Systolic blood pressure (mm Hg)	120 (9)	110 (8)	<0.001
Systolic hypertension (≥130 mm Hg) (*n*, %)	31 (11.6)	<5 (1.1)	<0.001
Diastolic blood pressure (mm Hg)	63 (6)	64 (6)	0.001
Cardiac measures			
Left ventricular mass indexed for height (g/m^2.7^)	37.69 (7.46)	35.59 (7.41)	0.003
Relative wall thickness (cm)	0.38 (0.06)	0.38 (0.06)	0.637
Left ventricular diastolic function (E/A)	1.94 (0.38)	1.90 (0.38)	0.350
Left ventricular filling pressure (E/e′)	4.89 (1.15)	4.81 (0.89)	0.875
Lifestyle factors			
Smoked cigarette ever (*n*, %)	109 (41)	130 (49.5)	0.776
Sedentary time (min/day)	468 (87)	484 (78)	0.023
LPA (min/day)	293 (70)	274 (62)	0.001
MVPA (min/day)	56 (30)	41 (24)	<0.001
Family history of H‐D‐C‐V (*n*, %)	82 (30.7)	85 (32.3)	0.322
Maternal socioeconomic status (*n*, %)			
Professional	22 (8.3)	16 (6.0)	0.213
Managerial and technical	103 (38.8)	99 (37.5)	
Skilled nonmanual	91 (34.3)	94 (35.6)	
Skilled manual	<7 (1)	<7 (1.7)	
Partly skilled	41 (15.3)	40 (15.1)	
Unskilled	7 (2.5)	11 (4.1)	

*Note*: The values are means (standard deviations) and *median (interquartile range) except for lifestyle factors in percentage. Differences between sexes were tested using Student's *t*‐test for normally distributed continuous variables, Mann–Whitney *U* test for skewed continuous variables, and chi‐square test for dichotomous variable and one‐way analysis of covariance for multicategory variable. A two‐sided *p*‐value <0.05 is considered statistically significant. *p*‐Value for sex differences.

Abbreviations: H‐D‐C‐V, hypertension, diabetes, high cholesterol and vascular disease; LPA, light physical activity; MVPA, moderate‐to‐vigorous physical activity.

### 3.2 Associations of ST, LPA, and MVPA with cardiac structure and function

Higher ST was associated with higher LVMI^2.7^ in females and the total cohort (Table 2). Higher MVPA was associated with higher LVMI^2.7^ in the total cohort. Higher ST and MVPA were associated with lower LVDF in males only (Table 2). Higher LPA was associated with none of the measures of cardiac structure and function in both males and females but was associated with higher LVDF in the total cohort (Table 2). ST, LPA, or MVPA had no significant associations with either RWT or LVFP in the total cohort, males, or females (Table 2).

**TABLE 2:** Sedentary time and physical activity in relation to cardiac structure and function at age 17 years based on sex category.

Predictor	Left ventricular mass index^2.7^		Relative wall thickness		Left ventricular diastolic function (E/A)		Left ventricular filling pressure (E/e′)	
	*β* (95% CI)	*p*‐Value	*β* (95% CI)	*p*‐Value	*β* (95% CI)	*p*‐Value	*β* (95% CI)	*p*‐Value
Male								
Sedentary time	−0.010 (−0.132 to 0.112)	0.868	−0.077 (−0.236 to 0.082)	0.340	−0.138 (−0.270 to −0.007)	**0.039**	0.021 (−0.130 to 0.175)	0.775
Light PA	−0.023 (−0.142 to 0.097)	0.711	−0.007 (−0.163 to 0.147)	0.917	0.112 (−0.015 to 0.240)	0.083	−0.004 (−0.153 to 0.143)	0.950
Moderate‐to‐vigorous PA	0.058 (−0.060 to 0.174)	0.335	−0.047 (−0.199 to 0.105)	0.542	−0.162 (−0.287 to −0.037)	**0.011**	−0.067 (−0.213 to 0.080)	0.370
Female								
Sedentary time	0.168 (0.040 to 0.197)	**0.010**	0.069 (−0.079 to 0.217)	0.360	0.014 (−0.113 to 0.139)	0.835	−0.012 (−0.134 to 0.111)	0.855
Light PA	0.076 (−0.042 to 0.197)	0.204	0.019 (−0.119 to 0.159)	0.777	0.086 (−0.034 to 0.205)	0.159	0.048 (−0.069 to 0.164)	0.422
Moderate‐to‐vigorous PA	0.107 (−0.003 to 0.222)	0.057	−0.031 (−0.162 to 0.099)	0.630	0.049 (−0.063 to 0.161)	0.386	−0.056 (−0.165 to 0.054)	0.318
Total cohort								
Sedentary time	0.093 (0.006 to 0.180)	**0.036**	−0.020 (−0.125 to 0.086)	0.712	−0.063 (−0.153 to 0.027)	0.169	−0.009 (−0.105 to 0.087)	0.850
Light PA	0.020 (−0.063 to 0.103)	0.635	−0.004 (−0.105 to 0.097)	0.936	0.099 (0.013 to 0.185)	**0.024**	0.023 (−0.069 to 0.114)	0.623
Moderate‐to‐vigorous PA	0.094 (0.014 to 0.174)	**0.021**	−0.025 (−0.121 to 0.072)	0.615	−0.049 (−0.131 to 0.034)	0.247	−0.083 (−0.171 to 0.005)	0.064

*Note*: Analyses were conducted using linear regression model and adjusted for age, high‐density lipoprotein cholesterol, low‐density lipoprotein cholesterol, triglyceride, glucose, insulin, heart rate, systolic blood pressure, high‐sensitivity C‐reactive protein, lean mass, fat mass, smoking status, family history of cardiovascular and metabolic disease, and sedentary time, light physical activity of moderate physical activity depending on the predictor. Analyses of the total cohort were further adjusted for sex. *β* is standardized regression coefficient and PA is physical activity. Statistically significant associations (in bold) were determined by a two‐sided *p*‐value <0.05.

Higher ST and MVPA were associated with higher LVMI^2.7^ in participants with optimal fat mass (Table 3), but not among those with high fat mass. Neither ST, LPA, nor MVPA had any significant associations with either RWT, LVDF, or LVFP based on fat mass stratification (Table 3). Higher ST and MVPA were associated with higher LVMI^2.7^ in participants with optimal lean mass but not among those with high lean mass (Table 4). Higher MVPA was associated with lower LVDF and LVFP among participants with high lean mass only (Table 4). Neither ST, LPA, nor MVPA had any significant associations with RWT based on lean mass stratification (Table 4).

**TABLE 3:** Sedentary time and physical activity in relation to cardiac structure and function at age 17 years based on fat mass category.

Predictor	Left ventricular mass index^2.7^		Relative wall thickness		Left ventricular diastolic function (E/A)		Left ventricular filling pressure (E/e′)	
	*β* (95% CI)	*p*‐Value	*β* (95% CI)	*p*‐Value	*β* (95% CI)	*p*‐Value	*β* (95% CI)	*p*‐Value
Optimal fat mass < 75th percentile								
Sedentary time	0.132 (0.029 to 0.235)	**0.012**	0.045 (−0.083 to 0.173)	0.490	−0.066 (−0.176 to 0.045)	0.230	−0.016 (−0.132 to 0.100)	0.785
Light PA	0.059 (−0.039 to 0.157)	0.298	0.036 (−0.085 to 0.158)	0.555	0.100 (−0.006 to 0.204)	0.064	−0.014 (−0.123 to 0.096)	0.806
Moderate‐to‐vigorous PA	0.106 (0.010 to 0.197)	**0.029**	−0.034 (−0.150 to 0.081)	0.557	−0.045 (−0.138 to 0.063)	0.460	−0.094 (−0.201 to 0.008)	0.072
High fat mass ≥ 75th percentile								
Sedentary time	0.045 (−0.156 to 0.245)	0.662	−0.153 (−0.355 to 0.049)	0.136	−0.005 (−0.164 to 0.155)	0.954	0.012 (−0.177 to 0.200)	0.942
Light PA	−0.005 (−0.197 to 0.188)	0.912	−0.096 (−0.291 to 0.097)	0.322	0.093 (−0.060 to 0.245)	0.233	0.076 (−0.107 to 0.258)	0.412
Moderate‐to‐vigorous PA	0.106 (−0.078 to 0.291)	0.292	0.031 (−0.155 to 0.216)	0.743	−0.043 (−0.187 to 0.101)	0.557	−0.025 (−0.200 to 0.150)	0.778

*Note*: Analyses were conducted using linear regression model and adjusted for age, sex, high‐density lipoprotein cholesterol, low‐density lipoprotein cholesterol, triglyceride, glucose, insulin, heart rate, systolic blood pressure, high‐sensitivity C‐reactive protein, lean mass, smoking status, family history of cardiovascular and metabolic disease, and sedentary time, light physical activity or moderate‐to‐vigorous physical activity depending on the predictor. *β* is standardized regression coefficient and PA is physical activity. Statistically significant associations (in bold) were determined by a two‐sided *p*‐value <0.05.

**TABLE 4:** Sedentary time and physical activity in relation to cardiac structure and function at age 17 years based on lean mass category.

Predictor	Left ventricular mass index^2.7^		Relative wall thickness		Left ventricular diastolic function (E/A)		Left ventricular filling pressure (E/e′)	
	*β* (95% CI)	*p*‐Value	*β* (95% CI)	*p*‐Value	*β* (95% CI)	*p*‐Value	*β* (95% CI)	*p*‐Value
Optimal lean mass < 75th percentile								
Sedentary time	0.103 (0.001 to 0.206)	**0.048**	0.014 (−0.111 to 0.139)	0.830	−0.031 (−0.140 to 0.079)	0.583	−0.013 (−0.132 to 0.105)	0.828
Light PA	−0.014 (−0.108 to 0.079)	0.762	0.054 (−0.061 to 0.168)	0.357	0.057 (−0.043 to 0.156)	0.264	0.027 (−0.080 to 0.135)	0.617
Moderate‐to‐vigorous PA	0.099 (0.005 to 0.194)	**0.038**	−0.050 (−0.165 to 0.065)	0.397	−0.009 (−0.109 to 0.092)	0.865	−0.055 (−0.163 to 0.053)	0.319
High lean mass ≥ 75th percentile								
Sedentary time	0.116 (−0.062 to 0.294)	0.201	−0.083 (−0.294 to 0.129)	0.485	−0.015 (−0.275 to 0.047)	0.163	0.013 (−0.182 to 0.155)	0.876
Light PA	0.195 (−0.005 to 0.396)	0.056	−0.173 (−0.411 to 0.065)	0.602	0.332 (0.148 to 0.515)	**<0.001**	0.013 (−0.180 to 0.207)	0.891
Moderate‐to‐vigorous PA	0.132 (−0.030 to 0.294)	0.109	0.074 (−0.119 to 0.266)	0.845	−0.150 (−0.296 to −0.003)	**0.046**	−0.176 (−0.332 to −0.021)	**0.026**

*Note*: Analyses were conducted using linear regression model and adjusted for age, sex, high‐density lipoprotein cholesterol, low‐density lipoprotein cholesterol, triglyceride, glucose, insulin, heart rate, systolic blood pressure, high‐sensitivity C‐reactive protein, fat mass, smoking status, family history of cardiovascular and metabolic disease, and sedentary time, light physical activity or moderate‐to‐vigorous physical activity depending on the predictor. *β* is standardized regression coefficient and PA is physical activity. Statistically significant associations (in bold) were determined by a two‐sided *p*‐value <0.05.

Higher ST and MVPA were associated with higher LVMI^2.7^ in both normal weight and participants who were overweight/obese based on the BMI category (Table 5). Higher ST was associated with lower RWT among participants who were overweight/obese (Table 5). Higher MVPA was associated with higher LVMI^2.7^ in normotensive participants only (Table 6). However, higher LPA was associated with higher LVDF in normotensive participants and lower LVFP among participants with elevated systolic BP/hypertension (Table 6).

**TABLE 5:** Sedentary time and physical activity in relation to cardiac structure and function at age 17 years based on BMI category.

Predictor	Left ventricular mass index^2.7^		Relative wall thickness		Left ventricular diastolic function (E/A)		Left ventricular filling pressure (E/e′)	
	*β* (95% CI)	*p*‐Value	*β* (95% CI)	*p*‐Value	*β* (95% CI)	*p*‐Value	*β* (95% CI)	*p*‐Value
Normal weight < 25 kg/m^2^								
Sedentary time	0.150 (0.051 to 0.250)	**0.003**	0.051 (−0.075 to 0.176)	0.488	−0.063 (−0.164 to 0.047)	0.247	−0.018 (−0.136 to 0.092)	0.744
Light PA	0.066 (−0.027 to 0.160)	0.161	0.050 (−0.067 to 0.168)	0.436	0.087 (−0.011 to 0.186)	0.083	0.011 (−0.096 to 0.119)	0.910
Moderate‐to‐vigorous PA	0.110 (0.017 to 0.203)	**0.021**	−0.024 (−0.142 to 0.093)	0.656	−0.026 (−0.125 to 0.073)	0.444	−0.052 (−0.159 to 0.055)	0.341
Overweight/obese ≥ 25 kg/m^2^								
Sedentary time	0.133 (0.041 to 0.230)	**<0.001**	−0.197 (−0.392 to −0.003)	**0.047**	0.038 (−0.147 to 0.217)	0.710	0.109 (−0.069 to 0.288)	0.258
Light PA	0.017 (−0.057 to 0.180)	0.555	−0.170 (−0.371 to 0.025)	0.086	0.119 (−0.063 to 0.302)	0.193	0.121 (−0.059 to 0.301)	0.186
Moderate‐to‐vigorous PA	0.123 (0.022 to 0.185)	**<0.001**	0.028 (−0.142 to 0.198)	0.746	−0.113 (−0.235 to 0.078)	0.279	−0.122 (−0.280 to 0.036)	0.128

*Note*: Analyses were conducted using linear regression model and adjusted for age, sex, high‐density lipoprotein cholesterol, low‐density lipoprotein cholesterol, triglyceride, glucose, insulin, heart rate, systolic blood pressure, high‐sensitivity C‐reactive protein, smoking status, family history of cardiovascular and metabolic disease, and sedentary time, light physical activity or moderate‐to‐vigorous physical activity depending on the predictor. *β* is standardized regression coefficient and PA is physical activity. Statistically significant associations (in bold) were determined by a two‐sided *p*‐value <0.05.

**TABLE 6:** Sedentary time and physical activity in relation to cardiac structure and function at age 17 years based on hypertensive status.

Predictor	Left ventricular mass index^2.7^		Relative wall thickness		Left ventricular diastolic function (E/A)		Left ventricular filling pressure (E/e′)	
	*β* (95% CI)	*p*‐Value	*β* (95% CI)	*p*‐Value	*β* (95% CI)	*p*‐Value	*β* (95% CI)	*p*‐Value
Normal/elevated systolic BP < 130 mmHg								
Sedentary time	0.088 (−0.002 to 0.179)	0.054	−0.013 (−0.119 to 0.098)	0.798	−0.071 (−0.159 to 0.025)	0.138	0.008 (−0.089 to 0.102)	0.876
Light PA	0.012 (−0.071 to 0.104)	0.785	−0.010 (−0.116 to 0.094)	0.839	0.114 (0.018 to 0.196)	**0.016**	0.039 (−0.054 to 0.131)	0.436
Moderate‐to‐vigorous PA	0.093 (0.014 to 0.181)	**0.022**	−0.029 (−0.130 to 0.071)	0.558	−0.047 (−0.119 to 0.050)	0.311	−0.086 (−0.166 to 0.010)	0.080
Systolic hypertension ≥ 130 mmHg								
Sedentary time	0.373 (−0.225 to 0.970)	0.200	−0.289 (0.900 to 0.421)	0.373	−0.012 (−0.972 to 0.949)	0.935	−0.070 (−0.327 to 0.187)	0.592
Light PA	0.146 (−0.271 to 0.563)	0.484	−0.203 (−0.690 to 0.357)	0.500	−0.145 (−0.788 to 0.499)	0.678	−0.093 (−0.180 to −0.014)	**0.033**
Moderate‐to‐vigorous PA	0.278 (−0.117 to 0.673)	0.157	0.071 (−0.439 to 0.552)	0.790	−0.168 (−0.866 to 0.531)	0.613	0.079 (−0.223 to 0.381)	0.604

*Note*: Analyses were conducted using linear regression model and adjusted for age, sex, high‐density lipoprotein cholesterol, low‐density lipoprotein cholesterol, triglyceride, glucose, insulin, heart rate, high‐sensitivity C‐reactive protein, lean mass, fat mass, smoking status, family history of cardiovascular and metabolic disease, and sedentary time, light physical activity or moderate‐to‐vigorous physical activity depending on the predictor. *β* is standardized regression coefficient and PA is physical activity. Statistically significant associations (in bold) were determined by a two‐sided *p*‐value <0.05.

## 4 DISCUSSION

In a large adolescent population, the present study revealed that device‐based measured ST, LPA, and MVPA have differing relationships with cardiac structure and function according to sex, overweight/obesity, body composition, and hypertensive status. Higher ST appears to consistently associate with higher LVMI^2.7^, while higher MVPA was paradoxically associated with higher LVMI^2.7^. Higher LPA was associated with higher LVDF in the total cohort and among participants who were normotensive and had high lean mass and lower LVFP among participants with elevated systolic BP/hypertension.

### 4.1 MVPA and cardiac structure and function

A daily 60 min of MVPA has been associated with cardiometabolic health benefits in youth and is thus recommended in public health guidelines.^9^ A meta‐analysis of 12 randomized controlled trials in 1266 children and adolescents concluded that short‐term exercise with a duration of 8–36 weeks did not reduce resting systolic and diastolic BP in children and adolescents.^42^ Similarly, a recent randomized clinical trial in youth that examined the effect of 60 min of aerobic training over 16 weeks reported no changes in BP across intervention and control groups after the intervention and 1‐year follow‐up.^43^ These findings^42^, ^43^ are important since higher BP has been associated with ventricular hypertrophy in youth.^2^, ^3^, ^5^ However, evidence on the role of MVPA on cardiac properties in adolescence is limited.^11^ It was observed that increased MVPA in males and among participants with high lean mass was associated with reduced LVDF. LVD dysfunction which usually culminates in increased LVFP has been associated with heart failure with preserved ejection fraction in adults.^6^ In youth, increased muscle mass and exercise may result in lower diastolic function complementary to increased cardiac mass which could suggest cardiac adaptation.^6^, ^34^ Moreover, among adolescents with high muscle mass MVPA was associated with lower LVFP. Together, these findings suggest that higher MVPA may benefit cardiac function and may attenuate the risk of cardiovascular disease progression.

Paradoxically, higher MVPA was associated with higher cardiac mass in the total cohort and among participants who were normal weight and overweight/obese using BMI categorization. This result was also observed among normotensive participants and those with optimal fat mass and lean mass but not with high fat mass, high lean mass, or elevated BP/hypertension. Previous studies in middle‐aged adults have reported conflicting relationships between increased MVPA and cardiac structure, specifically, increased LVMI.^12^, ^13^, ^14^ There were no associations between MVPA and RWT that reflects eccentric ventricular remodeling similar to an adult study report.^12^ Evidence from trained athletes suggests that eccentric hypertrophy is associated with endurance training whereas strength‐based training increases concentric hypertrophy.^44^ The paradoxical relationship between higher MVPA and higher LVMI in normotensive and optimal fat mass adolescent population may suggest physiological cardiac muscle increase which could complement the findings that higher MVPA was also associated with reduced LVDF and LVFP.^12^

In growing children and adolescents, it may be challenging to differentiate exercise‐related cardiac remodeling, pubertal‐related cardiac maturation, and subtle signs of cardiac structural disease like cardiomyopathies.^45^ A recent study revealed that a 1‐year early pubertal attainment, objectively assessed with age at peak height velocity, was associated with lower LVMI and RWT in 24‐year‐old males and females.^46^ This finding suggests that earlier puberty may not have a major impact on preclinical cardiovascular risk in early adulthood.^46^ In the present study, all participants had attained puberty by age 17 years of cardiac measures.^22^ In youth athletes, LV dilatation and mild concentric LV hypertrophy have been reported in cardiac adaptation to exercise training.^45^ This contrasts adults athletes' cardiac adaptation to exercise where less chamber dilatation and eccentric hypertrophy tend to occur.^45^ However, both youth and adult athletes exhibit similar increases in LV relaxation and improved LVDF.^45^ In the present study, higher MVPA was associated with higher LVMI but not RWT in the total cohort and lower LVDF in males. These findings in a resting state may suggest evidence of lower chamber dilatation, LV relaxation, and higher cardiac mass in adolescents, reflecting a subtle concentric remodeling.^34^, ^45^ Longitudinal studies are therefore warranted to examine the relationships between cumulative exposure to MVPA and cardiac structure and function changes during growth in youth.

### 4.2 LPA and cardiac structure and function

Evidence on device‐based measured LPA in relation to cardiac structure and function in youth is scarce. On average, most youths do not meet the recommended 60 min of MVPA per day^10^ as shown in the present study but accumulate on average more than 4 h of LPA per day. This present study showed that independent of ST, MVPA, cardiometabolic, and lifestyle factors, higher LPA had no relationship with altered cardiac structure either by sex, obesity, or body composition. Importantly, in the total cohort and among adolescents with high lean mass. Similarly, higher LPA was associated with higher LVDF; it was observed that in normotensive adolescents, higher LPA was associated with higher LVDF and lower LVFP in hypertensive participants. It is known that decreased LVDF and increased LVFP are precursors of heart failure with preserved ejection fraction,^6^ and this present finding suggests that higher LPA may attenuate the cardiac dysfunction sequelae of elevated BP/hypertension in adolescents. Nonetheless, more studies and clinical trials are needed to examine the longitudinal effect of LPA on cardiac function in youth, and whether LPA could be prescribed to enhance better cardiac function in early life.

### 4.3 ST and cardiac structure and function

Sedentary behavior assessed from self‐report has been associated with obesity and adverse cardiometabolic health in youth, but device‐based measured ST was not associated with adiposity and other cardiovascular risks.^9^, ^15^ Moreover, evidence on device‐based measured ST in relation to cardiac function and structure in adolescents is lacking.^9^ This present study is one of the first and largest studies in this age group to investigate the association between higher ST and cardiac indices. A consistent and positive association between higher ST and LVMI in females, normal weight, and overweight/obese participants, and in the total cohort independent of LPA, MVPA, cardiometabolic, and lifestyle factors was observed. In the present study, females accumulated an additional 15‐min ST than males and have nearly twice higher fat mass than their male counterparts. Considering the significant amount of time spent in ST, the association of higher ST with higher LVMI may result in a higher cardiac mass when compared to the MVPA‐associated LVMI increase. In the unstandardized regression analysis (data not shown) of the total cohort, each 1‐min higher ST was associated with a 0.008 g/m^2.7^ higher LVMI, whereas each 1‐min higher MVPA was associated with 0.025 g/m^2.7^ higher LVMI. On average, participants had 476 min of ST which may correspond to a 3.8 g/m^2.7^ of LVMI. On the other hand, participants had an average of 49 min of MVPA which may correspond to a 1.2 g/m^2.7^ of LVMI. The ST‐related increase in LVMI may be clinically significant since among adults, a ~5 g/m^2^ higher LVMI may correspond to a 7%–20% increase in CVD morbidity and mortality.^47^

### 4.4 Strength and limitation

Using a well‐phenotyped large birth cohort (ALSPAC) with extensive assessments during adolescence, the present study investigated the relationship of time spent in ST, LPA, and MVPA, with cardiac structure and function. A comprehensive covariate adjustment for device‐measured body composition such as fat mass, lean mass, and lifestyle factors including smoking and family history of cardiovascular and metabolic diseases provides an understanding of the independent relationships of movement behavior with cardiac indices. The participants were mostly Caucasian with minimal geographic variations; thus, these findings may not be generalizable to other ethnicities. The cross‐sectional design of the study precludes causal inferences; thus, longitudinal studies and clinical trials are warranted. The accelerometer was worn for 7 days, which may not be sufficient to reveal the habitual lifestyle of adolescents. The possibility of a Hawthorne effect cannot be excluded where participants modify their behavior based on their awareness of being observed. Nonetheless, the device‐measured PA and ST are superior to self‐reported lifestyle behavior which is fraught with recall and report bias.^15^ Variables such as smoking status were retrieved by validated ALSPAC questionnaires; however, recall bias may not be excluded.

## 5 CONCLUSION

Among adolescents, higher ST was associated with higher LVMI in adolescents which is threefold higher than MVPA‐associated LVMI increase. Higher LPA was associated with higher LVDF and lower LVFP in adolescents. Efforts targeted at decreasing ST and increasing LPA may enhance cardiac structural and functional health in youth. Longitudinal studies and clinical trials examining the effect of device‐based measured movement behavior on cardiac structure and function in youth are warranted.

## AUTHOR CONTRIBUTIONS

Dr A.O. Agbaje had full access to all the data in the study and take responsibility for the integrity of the data and the accuracy of the data analysis. A.O. Agbaje contributed to concept and design, acquisition of data from ALSPAC, analysis, or interpretation of data, drafting of the manuscript, critical revision of the manuscript for important intellectual content, statistical analysis, and obtained funding. ALSPAC contributed to data collection. This publication is the work of the author and A.O. Agbaje will serve as guarantor for the contents of this paper.

## FUNDING INFORMATION

The UK Medical Research Council and Wellcome (Grant ref: 217065/Z/19/Z) and the University of Bristol provide core support for ALSPAC. The British Heart Foundation grant (CS/15/6/31468) funded blood pressure and Actigraph activity monitoring device measurement. The Medical Research Council grant (MR/M006727/1) supported smoking data collection. A comprehensive list of grant funding is available on the ALSPAC website (http://www.bristol.ac.uk/alspac/external/documents/grant‐acknowledgements.pdf); this publication is the work of the authors, and AOA will serve as guarantor for the contents of this paper. Dr Agbaje research group (UndeRstanding FITness and Cardiometabolic Health In Little Darlings ( *urFIT‐child* )) was specifically funded by the Jenny and Antti Wihuri Foundation (Grant no: 00180006); the North Savo regional and central Finnish Cultural Foundation (Grants no: 65191835, 00200150, and 00230190), Orion Research Foundation sr, Aarne Koskelo Foundation, Antti and Tyyne Soininen Foundation, Paulo Foundation, Paavo Nurmi Foundation, Yrjö Jahnsson Foundation (Grant no: 20217390), and the Finnish Foundation for Cardiovascular Research (Grant no: 220021). The funders had no role in the design and conduct of the study; collection, management, analysis, and interpretation of the data; preparation, review, or approval of the manuscript; and decision to submit the manuscript for publication.

## CONFLICT OF INTEREST STATEMENT

None.

## Data Availability

The informed consent obtained from ALSPAC participants does not allow the data to be made freely available through any third‐party maintained public repository. However, data used for this submission can be made available on request to the ALSPAC Executive. The ALSPAC data management plan describes in detail the policy regarding data sharing, which is through a system of managed open access. Full instructions for applying for data access can be found here: http://www.bristol.ac.uk/alspac/researchers/access/. The ALSPAC study website contains details of all the data that are available at: http://www.bristol.ac.uk/alspac/researchers/our‐data/.
